# Balancing Near-Field Enhancement and Hot Carrier Injection: Plasmonic Photocatalysis in Energy-Transfer Cascade Assemblies

**DOI:** 10.1021/acsphotonics.3c00733

**Published:** 2023-09-06

**Authors:** Yoel Negrín-Montecelo, Adbelrhaman Hamdeldein Ahmed Geneidy, Alexander O. Govorov, Ramon A. Alvarez-Puebla, Lucas V. Besteiro, Miguel A. Correa-Duarte

**Affiliations:** † Department of Physical and Inorganic Chemistry, Universitat Rovira i Virgili, Carrer de Marcel•lí Domingo s/n, 43007 Tarragona, Spain; ‡ CINBIO, University of Vigo, Campus Universitario de Vigo, Lagoas Marcosende, 36310 Vigo, Spain; § Department of Physics and Astronomy, Ohio University, Athens, Ohio 45701, United States; ∥ ICREA, Passeig Lluís Companys 23, 08010 Barcelona, Spain; ⊥ Southern Galicia Institute of Health Research (IISGS) and Biomedical Research Networking Center for Mental Health (CIBERSAM), Universidade de Vigo, 36310 Vigo, Spain

**Keywords:** hot electrons, field enhancement, quantum dots, nanostructures, photocatalysis, plasmonics

## Abstract

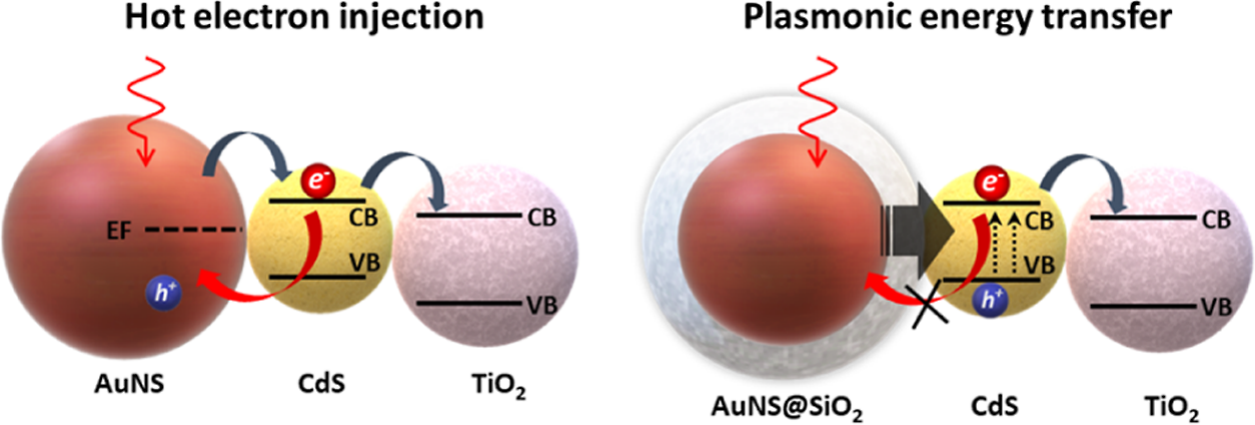

Photocatalysis stands as a very promising alternative to photovoltaics in exploiting solar energy and storing it in chemical products through a single-step process. A central obstacle to its broad implementation is its low conversion efficiency, motivating research in different fields to bring about a breakthrough in this technology. Using plasmonic materials to photosensitize traditional semiconductor photocatalysts is a popular strategy whose full potential is yet to be fully exploited. In this work, we use CdS quantum dots as a bridge system, reaping energy from Au nanostructures and delivering it to TiO_2_ nanoparticles serving as catalytic centers. The quantum dots can do this by becoming an intermediate step in a charge-transfer cascade initiated in the plasmonic system or by creating an electron–hole pair at an improved rate due to their interaction with the enhanced near-field created by the plasmonic nanoparticles. Our results show a significant acceleration in the reaction upon combining these elements in hybrid colloidal photocatalysts that promote the role of the near-field enhancement effect, and we show how to engineer complexes exploiting this approach. In doing so, we also explore the complex interplay between the different mechanisms involved in the photocatalytic process, highlighting the importance of the Au nanoparticles’ morphology in their photosensitizing capabilities.

## Introduction

TiO_2_ has been a pioneering material that presents very interesting physical and chemical properties such as outstanding chemical stability, low toxicity, low cost, good supportability on a variety of substrates, and high photocatalytic activity under ultraviolet (UV) excitation.^1^ As a semiconductor, it absorbs photons with sufficient energy to have an electron transition from its valence band (VB) to its conduction band (CB), thus generating an electron–hole pair. These charges can create reactive species in solution that can be involved in oxidation or reduction processes.^2^ Due to the good alignment of its VB and CB with the redox potential of key reactions, there have been many different implementations of TiO_2_ nanoparticles as a photo-active component in applications such as air and water purification, hydrogen evolution, sterilization, and the formation of self-cleaning devices and anti-fogging surfaces.^1,3−6^ Nevertheless, the main disadvantage of the use of TiO_2_ as a photocatalyst is related to its large band gap (3.2 eV for anatase), leading to excitations restricted to the UV segment of the solar spectrum, thus rendering it a relatively inefficient material to exploit solar radiation.^7^

The potential of this wide band gap semiconductor for photocatalysis has led to the pursuit of different strategies to overcome its limitations in terms of external quantum efficiency, including morphological modifications (increasing surface area or porosity) and chemical transformations (nitrogen doping and co-doping with other elements, surface complexation, and sensitization by inorganic complexes or organic dyes), aiming at tailoring the band gap of TiO_2_.^8−11^ The ability of plasmonic nanoparticles (PNPs) to harvest visible and NIR light can be used to extend the spectral range usable by a nearby semiconductor, increasing the photocatalytic activity of the hybrid under sunlight or other broad-spectrum illumination. In this framework, departing from traditional thermal catalysis, the plasmonic activation of TiO_2_ can be explained through two different mechanisms: hot-electron injection (HEI) and near-field effects allowing plasmonic energy transfer (PET). Each of them has its own advantages and drawbacks, but detailed knowledge of how to balance and exploit them optimally is still lacking.^12−16^ One should also note that photoheating can also contribute to the catalysis,^17,18^ but in this study we focus on the mechanisms that more clearly separate plasmonic photocatalysis from traditional thermal catalysis. Consequently, we have used experimental conditions that avoid the contribution of photoheating, so as to discern the balance between HEI and PET: we clamped the temperature of the sample to a homogeneous low value with a thermal bath and magnetic stirring,^19^ and we worked with light intensities that can only lead to a minuscule local temperature rise.^20−22^ Of course, exploiting the HEI and PET mechanisms offered by plasmonic nanostructures is of use beyond photosensitizing semiconductors, e.g., driving photocatalysis directly,^23,24^ controlling the photogrowth of NPs with special shapes,^25−27^ allowing chiral plasmonic photochemistry,^28,29^ or for SERS applications.^30,31^

After the absorption of light, the localized surface plasmon resonance (LSPR) created on a metal NP produces a population of excited electrons that oscillate in resonance with the incoming field. A small population of these electrons have higher kinetic energies, referred to as “hot electrons,” and can participate in the HEI process. That high energy allows them to leave the metal and be transferred to a nearby semiconductor.^32^ The overall capability of a PNP to induce HEI depends on several factors, including the size, shape, and composition of the metal NP, as well as the type of interface connecting metal and semiconductor.^11,33,34^ However, even in scenarios conducive to an efficient HEI across the interface, this effect is partially balanced by the back-transfer of injected carriers to the metal, where their kinetic energies are rapidly shared with the other electrons in the Fermi sea. This deleterious effect is understood to be reduced when creating a Schottky barrier between metal and semiconductor, blocking electrons at the CB edge to return to the metal. Importantly, the balance between these two effects depends on the concentration of the PNPs and previous reports have shown that quantities exceeding a certain metal concentration threshold induce a decrease in the net photocatalytic efficiency.^11,14,35,36^

The PET mechanism can be understood as arising from a light concentration effect at the semiconductor through the enhanced electric near-field induced by the PNPs,^37−39^ or trough models such as plasmon-induced resonance energy transfer (PIRET), a non-radiative dipole–dipole energy transfer between the metal and the semiconductor.^12,13,40^ In either case, PET only occurs when there is spectral overlap between the plasmonic modes of the PNPs and the absorption band of the semiconductor. In contrast to HEI, PET processes do not involve charge transfer between the metal and other elements and can occur in geometries where the plasmonic sensitizer and the semiconductor are separated by an insulating material.^13,35^

Another approach capable of enhancing the photocatalytic properties of TiO_2_ is the combination of TiO_2_ with other semiconductors with narrower band gaps that allow the absorption of lower-energy photons from the visible region.^41−44^ Moreover, if the CB of the second semiconductor lies at higher energies than that of TiO_2_, it is beneficial for enhancement of the charge separation, in a cascade-like process. In this work, we have used CdS quantum dots (CdS-QDs) to accrue these advantages. Besides being cheap and easy to synthesize, the absorption of CdS partially overlaps with the plasmonic resonance of small Au NPs, which makes it an interesting candidate for creating plasmonic-semiconductor hybrids,^39,45^ both in colloidal suspension^37^ and creating planar metamaterials.^46^ While the photogenerated electrons in the CdS CB are transferred to the TiO_2_, the photogenerated holes remain in the CdS VB. In this manner, the charge recombination probability is also reduced as a result of the separation effect, to the benefit of the energy conversion efficiency and overall photocatalytic activity.^47,48^ The addition of PNPs to the TiO_2_/QDs assemblies can further enhance the overall photocatalytic performance of the composites by increasing the charge carriers transferred.^49−52^ One can also consider the QDs as a “bridge” between the PNPs and the TiO_2_ NPs, in terms of both HEI and, perhaps more importantly, PET due to the more significant overlap between the PNP and QD spectra. This situation is in contrast with that of a metallic catalyst in interaction with the PNPs, in which PET is possible at broader wavelength ranges.^53−55^

Herein, we report a layer-by-layer (L-B-L) synthesis strategy for the hierarchical assembly of either Au PNPs or core–shell Au@SiO_2_ PNPs with CdS-QDs and TiO_2_ NPs onto submicrometric silica beads (SiO_2_). The SiO_2_ shell over the PNP will block charge transfer events to and from the metal NPs. These hybrid photocatalysts will be a test bench onto which to explore the outcomes of making either HEI or PET the dominant energy transfer mechanism and evaluate what design leads to greater efficiencies. On these lines, we will also explore the effect of the L-B-L deposition order over the final photocatalytic effects, supporting our description of the hybrid photocatalysts’ operation and offering additional insights into the engineering of successful photocatalytic complexes.

## Results and Discussion

**Figure 1:**
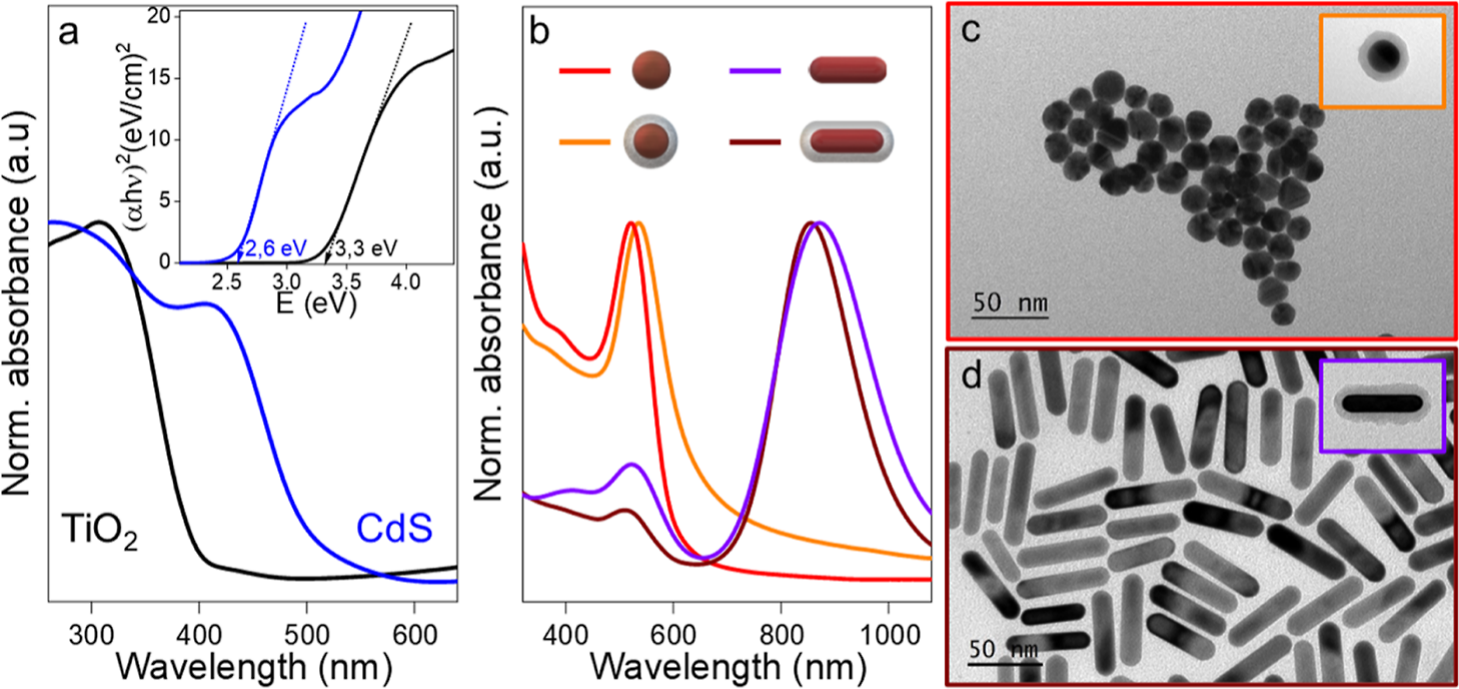
(a) Absorbance spectra resulting from semiconductor absorption and band gap calculation (Tauc plot) of TiO_2_ NPs (black) and CdS-QDs (blue). (b) Normalized absorbance spectra of AuNSs (red), AuNS@SiO_2_ (orange), AuNRs (brown), and AuNR@SiO_2_ (purple). (c,d) TEM images of the AuNS and AuNR, respectively (those of the silica-coated particles are inserted in the upper right corner of each panel).

As illustrated in Figure 1a, the commercial TiO_2_ NPs and the prepared CdS-QDs have band gaps of 3.3 and 2.6 eV, respectively, as calculated based on the experimental extinction spectra by means of Tauc’s plot. In this manner, the addition of CdS alone in a hybrid catalyst with TiO_2_ can enhance the photo-absorption of light in the visible region.

**Figure 2:**
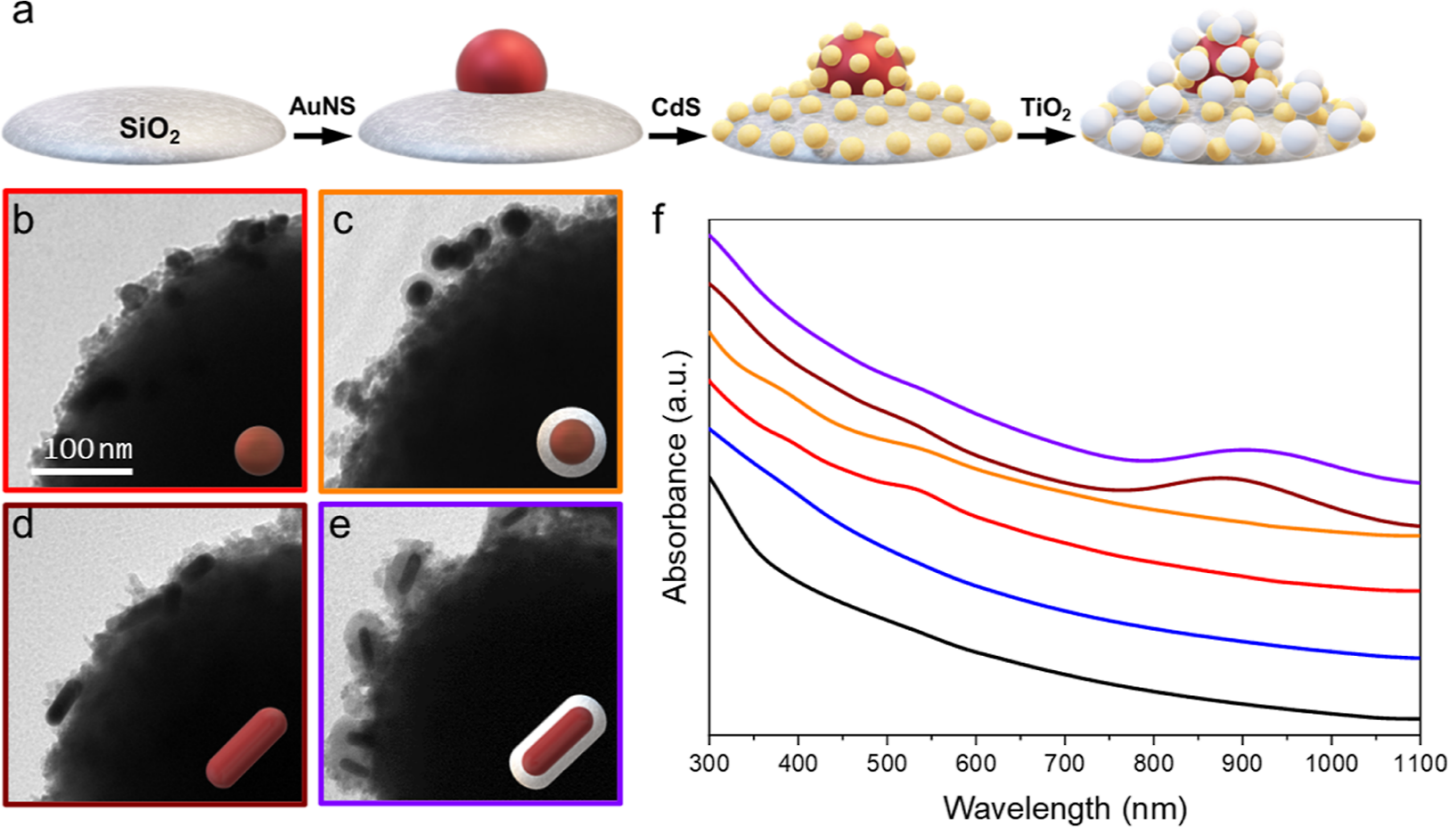
(a) Scheme of the layer-by-layer assembly of the hybrid photocatalysts. In the first step, the chosen PNPs are adsorbed onto the surface of SiO_2_ beads, and subsequently, the layers of CdS-QDs and TiO_2_ NP are added. (b–e) TEM images and (f) vertically offset absorbance spectra of the hybrid photocatalysts formed with (b) AuNS/CdS/TiO_2_ (red line), (c) AuNS@SiO_2_/CdS/TiO_2_ (orange line), (d) AuNR/CdS/TiO_2_ (brown line), and (e) AuNR@SiO_2_/CdS/TiO_2_ (purple line). Black and blue spectra correspond to hybrid assemblies with TiO_2_ and CdS/TiO_2_, respectively.

We have synthesized four different types of Au NPs to integrate them onto the TiO_2_/CdS-based hybrid nanostructures reported herein. These four systems are created in order to explore two different aspects of the PNP contribution: (i) choosing the wavelength of the main LSPR, and (ii) investigating whether they can contribute through HEI. First, we created naked and silica-coated gold nanospheres (AuNS and AuNS@SiO_2_), with their main LSPR in the visible spectrum, displaying plasmonic signatures centered at 523 and 535 nm, respectively (red and orange spectra, Figure 1b) and with the AuNS diameter fixed at 23 nm (Figure 1c). Second, we created naked and silica-coated gold nanorods (AuNR and AuNR@SiO_2_), with their main LSPR in the near-IR region, with longitudinal plasmon bands centered at 856 nm and 871 nm, respectively (brown and purple spectra, Figure 1b), and with an aspect ratio of 4.15 (54 × 13 nm, Figure 2d). In the case of silica-coated nanoparticles, the maximum absorption is shifted to higher wavelengths, with respect to the original AuNP, as a result of the refractive indices increasing once the AuNPs are screened by the SiO_2_ shell.^56^ In both Au@SiO_2_ NPs, the SiO_2_ shell is homogeneous, with a thickness of 8 nm (Figure S1). In this manner, we will explore four distinct scenarios by combining two different well-differentiated plasmonic signatures with two different dominant photosensitization mechanisms. Accordingly, the naked resonators will be in direct contact with the QDs/TiO_2_ assemblies showing a dominant HEI mechanism while the silica-coated resonators will show a pure PET mechanism due to their isolated silica shell. Moreover, by utilizing both AuNS and AuNR, we can compare plasmonic excitation from different regions of the electromagnetic spectrum.

As mentioned above, the different Au nanoparticles are integrated into hybrid photocatalysts. For this purpose, silica beads with a diameter of 525 nm exposed to a positively charged solution of polyelectrolyte have been used as supports for the adsorption of the negatively charged PNPs, followed by the adsorption of the CdS-QDs and, finally, another layer with 5 nm TiO_2_ NPs (Figure 2a). This layer-by-layer protocol has been previously used for the assembly of PNP and semiconductors, permitting tight control over the composition and functionality of the final structure.^11,35,57^ For comparison purposes, the PNP/CdS/TiO_2_ hybrids formed present the same Au/Cd/Ti molar ratio (0.0711/0.121/1, determined by ICP-OES, Table S1). Moreover, the catalysts present high homogeneity and colloidal stability (Figure 2b–e). With respect to the optical properties (Figure 2f), the hybrids display a strong absorption under 500 nm as a consequence of QDs and TiO_2_ absorption in the visible and UV regions, respectively. Additionally, an important scattering contribution of the SiO_2_ beads is perceived at lower wavelengths, with a long tail covering the visible spectrum and part of the NIR. Over it, the LSPR signatures of the naked and silica-coated nanoparticles are discernible (Figure 2f) with a slight red shift with respect to the free particles in solution as a consequence of the larger effective refractive index created by the materials surrounding the PNPs.^58,59^

**Figure 3:**
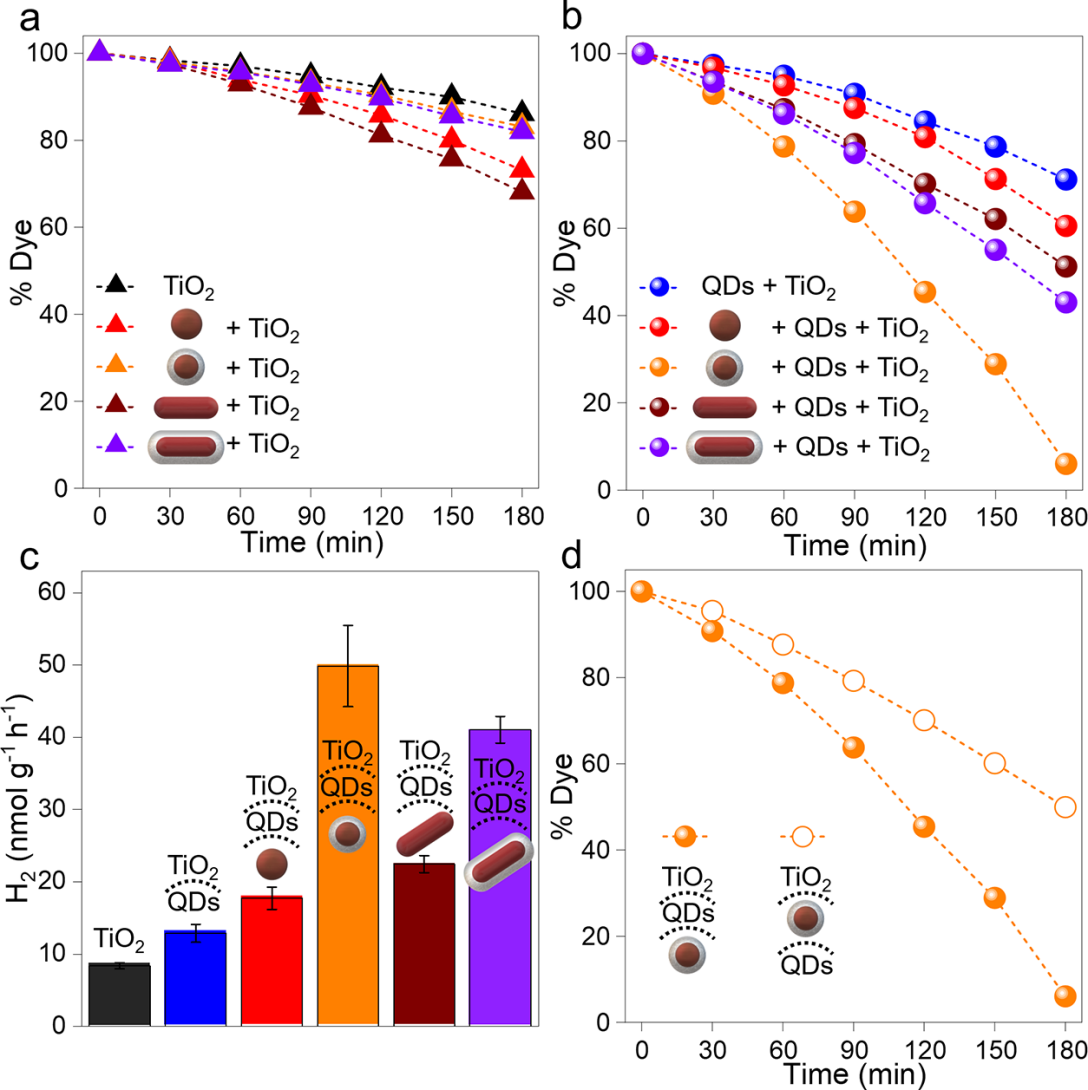
Photocatalytic degradation of RhB in the presence of the hybrids with TiO_2_ (a) and with QDs and TiO_2_ (b). (c) Photocatalytic hydrogen generation assisted by formic acid in the presence of different hybrids (nanomoles of H_2_ per mg of catalyst in 1 h of reaction). (d) Photocatalytic degradation of RhB in the presence of the catalyst composed by AuNS@SiO_2_, QDs, and TiO_2_ by changing the order of the L-B-L assembly (AuNS@SiO_2_/QDs/TiO_2_, orange circles; QDs/AuNS@SiO_2_/TiO_2_, white circles). In all panels, the catalyst with TiO_2_ alone is represented in black and the catalyst with QDs and TiO_2_ alone is represented in blue; catalyst with AuNSs, AuNS@SiO_2_, AuNRs, and AuNR@SiO_2_ is represented in red, orange, brown, and purple, respectively (triangles for control experiments without QDs and circles for hybrids with QDs and TiO_2_). *P* = 1.0 atm, *T* = 25 °C, and λ ∈ [350,2400] nm.

**Figure 4:**
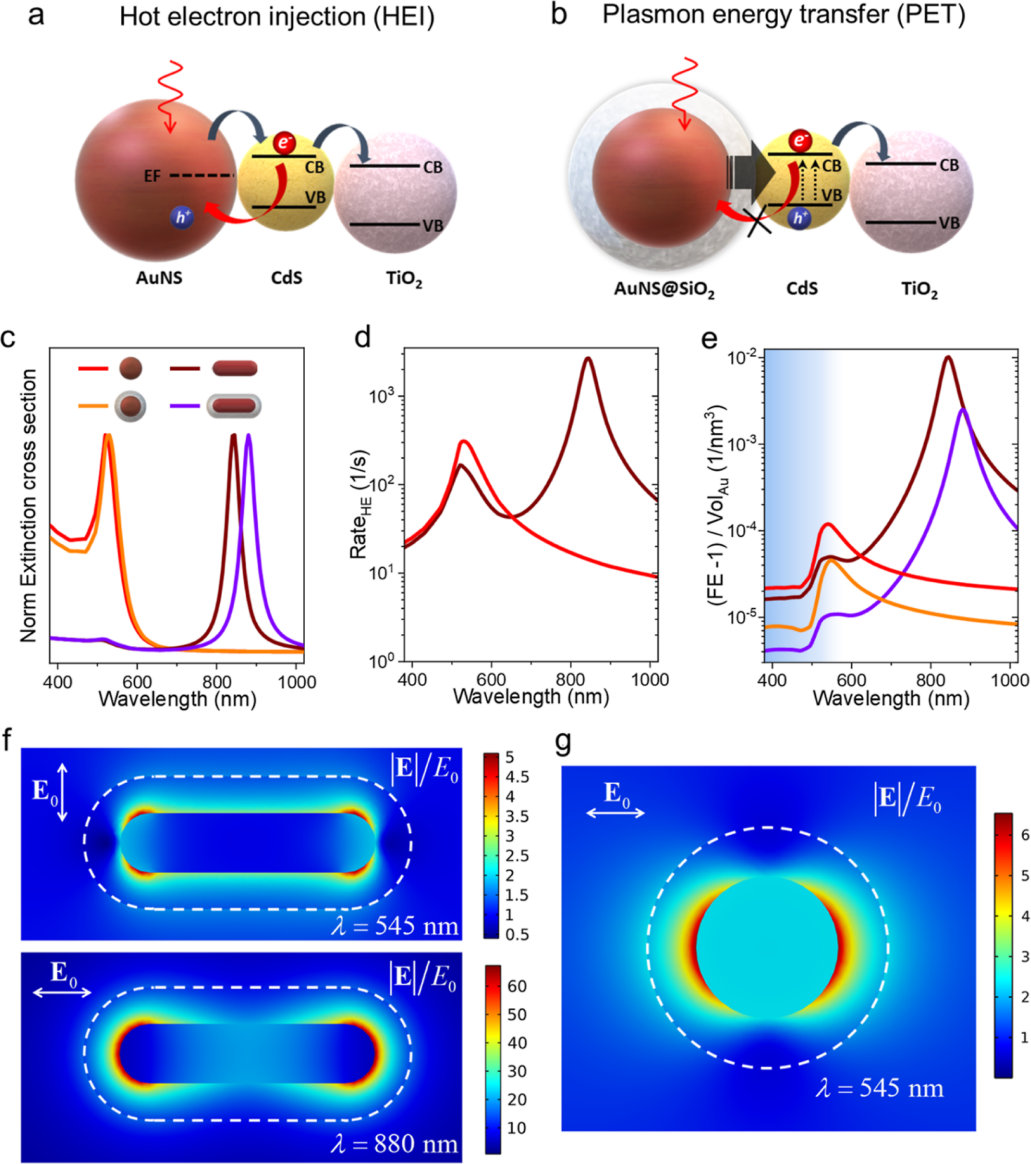
(a) Representation of the HEI mechanism in the hybrid composed of AuNS/CdS-QDs/TiO_2_ (upper panel) and PET in the hybrid composed of AuNS@SiO_2_/CdS-QDs/TiO_2_ (lower panel). (b) Theoretical extinction cross sections of AuNS, AuNS@SiO_2_, AuNR, and AuNR@SiO_2_. (c) Rate of intraband hot carrier excitation for naked AuNSs and AuNRs. (d) Average field enhancement (eq 1) and (e) average field enhancement, referenced to zero and normalized by the Au volume of each particle. The blue region in panel (e) indicates the spectral region with non-zero QD absorption. In all figures, AuNSs, AuNS@SiO_2_, AuNRs, and AuNR@SiO_2_ are represented in red, orange, brown, and purple, respectively. (f,g) Electric field maps for the transversal and longitudinal modes of AuNR@SiO2 (f) and for the plasmonic mode of AuNS@SiO_2_ (g). The external boundary of the SiO_2_ layer is highlighted with a dashed white line.

The photocatalytic efficiency of the synthesized nanocomposites has been evaluated through the photodegradation of rhodamine B (RhB) and formic acid dehydrogenation, which serve as model reactions. In this manner, the degradation of RhB in the presence of the hybrids was monitored by following the decrease in the absorption maximum of this dye (λ_max_ = 554 nm) as a function of time, using a solar simulator with emission ranging from 350 nm to 2400 nm. First, the photocatalytic experiment was performed using the catalyst without the QD layer (Figure 3a), providing us with a baseline of the TiO_2_ photoactivity. After 3 h of reaction, only 13.90% of degradation was observed using the SiO_2_ beads functionalized with TiO_2_ NPs alone (black line, Figure 3a) as a result of the direct photoexcitation of the semiconductor with the small fraction of UV photons. In the case where we added naked AuNS and AuNR, the degradation of the dye was increased to 26.89% and 31.91%, respectively, during the same period of time (red and brown lines, Figure 3a) as a result of the combined effect of HEI and PET mechanisms by the PNP. The increased efficiency for the HEI with AuNRs with respect to AuNSs for the same Au/Ti molar ratio is due to the former having more intense local fields around the tips of the PNPs for sharper anisotropies as well as the presence of the main resonance at longer wavelengths (see Figure 4c).^11,60^ Unsurprisingly, the addition of silica-coated gold nanoparticles does not have a major influence on the final degradation with respect to the system without PNPs (orange and purple lines, Figure 3a) as only the PET mechanism can occur in these systems, and the spectral overlap between the plasmonic modes and the TiO_2_ absorption is very small.

When the same experiment was performed using the catalysts with the QD layer and in the absence of AuNP, the degradation of RhB increased from 13.90% (black line, Figure 3a) to 28.8% (blue line, Figure 3b). The enhanced photocatalytic activity can be attributed to a more effective response of the catalyst with QDs in the visible region.^41−44^ As mentioned above, we expect this effect to increase when adding PNPs as the photosensitizers. With the addition of AuNSs and AuNRs onto the assemblies, the degradation increases to 39.48% and 48.77%, respectively (red and brown lines, Figure 3b). Interestingly, the use of catalysts with AuNS@SiO_2_ and AuNR@SiO_2_ leads to 93.96% and 56.97% of degradation, respectively. We discuss these differences below, with the support of computational results.

We reproduced the same general trends with an additional model reaction, measuring the photocatalytic hydrogen generation assisted by the degradation of formic acid in the presence of the hybrid nanomaterials as photocatalysts (Figure 3c).^33,61^ This experiment produces the same trend reported for the photodegradation of RhB. In this manner, the photogenerated hydrogen increases from 12.88 nmol g^–1^ h^–1^, when using assemblies without Au (blue column, Figure 3c), to 17.72 nmol g^–1^ h^–1^ and 22.45 nmol g^–1^ h^–1^ for naked AuNSs and AuNRs (red and brown columns, Figure 3c), respectively. In the presence of the silica-coated AuNS@SiO_2_ and AuNR@SiO_2_, the photogenerated hydrogen increases to 49.86 nmol g^–1^ h^–1^ and 41.05 nmol g^–1^ h^–1^, respectively.

Finally, we investigated the influence of the assembly order on the layer-by-layer synthesis of these nanohybrids. To achieve this, we selected the catalyst, among the ones we studied, with the highest photocatalytic efficiency, modified the hierarchy in which the layers were assembled, and then performed the photocatalytic degradation of RhB under similar conditions. As we can see in Figure 3d, when we deposited the AuNS@SiO_2_ NPs in the middle layer between the CdS-QDs and the TiO_2_ NPs, the final degradation decreases from 93.96 to 50.04% (full and hollow orange circles, respectively). These results support the interpretation that the CdS-QDs serve as an energy pathway between PNP and TiO_2_, a function that they fulfill when they are between, and in direct contact with, the PNP and the TiO_2_ NPs. In other terms, depositing the QDs after the PNP greatly increases the number of CdS-QDs that are close to both PNP surfaces and TiO_2_ NPs. By first depositing the QDs, many of them will be either under the PNP and thus not in close contact with TiO_2_ NPs, or near TiO_2_ NPs, but far from the PNP-enhanced near field.

Turning now our attention to the phenomena underlying the reaction in the different configurations, we can explain the increased photocatalytic activity of the PNP-loaded hybrids by taking into consideration the main photoactivation mechanism of each system. In the hybrids with naked AuNP (Figure 4a), the plasmonic excitation produces a population of “hot electrons” that can be injected from the CB of the Au NPs to the CB of CdS-QDs and, finally, transferred to the CB of the TiO_2_ NPs. It is important to remember, however, that in this configuration, the PNP also acts as a recombination center, reducing the overall electron events contributing to the reaction.^14,35,36,62^ In the case of silica-coated nanoparticles (Figure 4b), the electromagnetic field enhancement produced by the LSPR increases the charge separation in the CdS-QDs near the AuNS@SiO_2_. This effect, which should contribute in the case of naked PNP, is now dominant because HEI, as well as charge back-transfer, is blocked by the SiO_2_ layer. Now, electrons excited within the QDs can be transferred from the CB of the QDs to the CB of the TiO_2_ NPs. Again, it is important to note that the silica layer prevents charge transfer from the semiconductors to the metal. To further support this interpretation of the results in Figure 3b,c, we performed additional photocatalytic experiments using a band-pass filter that allows passage only to light with wavelengths over 700 nm (see Figure S2). When using AuNRs@SiO_2_ hybrid photocatalysts, the activity drops to virtually nothing, as we are not driving the transversal plasmonic mode capable of exciting the QDs through its enhanced near-field, whereas when using naked AuNRs, we see a reduced but still significant degradation, as we lose the PET effect and the HEI of the transversal mode but retain the HEI of the main AuNR mode at longer wavelengths. Importantly, in this latter case, electron back-transfer still hinders the photoreaction, limiting the otherwise large capability of the AuNRs of exciting hot electrons, as discussed below.

To extend our discussion on the role that the different photosensitization mechanism has in these hybrids, we complement the experimental results with computational simulations of AuNR and AuNS modeled using the shapes and sizes of the experimental samples. First, upon comparing the computed extinction cross sections of the PNP (Figure 4c) with their experimental absorbance (Figure 1b), we see that the theoretical models reproduce well the optical properties of the samples. If we look at the potential contribution of the different energy transfer mechanisms (Figure 4a), we can distinguish different relevant aspects to discuss. In Figure 4d, we can see the rates of excitation of intraband hot carriers at the surface of the PNPs. At this point, only the results for naked AuNSs and AuNRs are included because by coating the PNP with SiO_2_, we avoid the transfer of hot electrons between the metal and the environment. In this plot, it is apparent that the stronger longitudinal plasmonic mode of the AuNR dominates in exciting hot electrons, over both its transversal mode and the AuNS plasmon. It also underscores that allowing hot carrier injection as a photocatalytic mechanism allows the sensitization of the system to photons with lower energies, up to wavelengths in the near-IR with the AuNR studied herein. This comes at the cost, however, of allowing carrier recombination through electron back-transfer to the metal.^63^ Qualitatively, this explains the modest improvement seen in Figure 3a when using AuNR (brown triangles) over AuNS (red triangles). In addition, with the naked AuNP, the excitation of CdS-QDs and TiO_2_ through the near-field also occurs, to the extent that their absorption bands spectrally overlap with the plasmon resonances, which is when we consider the SiO_2_-coated AuNP for isolating this contribution.

Then, considering energy transfer through near-field interactions between AuNP and the environment, we present in Figure 4e the volume-averaged field enhancement (FE) in the space available around the AuNP or its SiO_2_ coating. Here, it is clear that the strongest contribution could come from the longitudinal resonances of the AuNR, but the hybrid photocatalyst cannot take the energy stored in the plasmonic near-field at these wavelengths because the semiconductors do not absorb in this part of the spectrum. In contrast, the near-field enhancement at higher energies by the AuNP can enhance the absorption rates of the QDs/TiO_2_ assemblies. Therefore, when considering the effects driving the photodegradation of RhB, we should focus on the FE in wavelengths under 550 nm. At these wavelengths, our computational results predict that the contribution of AuNS@SiO_2_ would be larger than that of AuNR@SiO_2_ on a per-particle basis (see Figure S3). This is also the case when we consider the contribution to the local FE on a “per-atom” basis (see Figure 4e), which can be done by comparing the “excess” average field enhancement normalized by the AuNP volume. This conclusion provides an explanation for the experimental results in Figure 3b,c, where these are precisely the hybrids showing a faster photodegradation. This arises from the fact that we are considering colloidal NPs so that the anisotropy of the AuNR implies that only a fraction of the ensemble will see its high-energy, transversal plasmonic mode excited at a given time, whereas the spherical symmetry of the AuNS guarantees a homogeneous performance in this regard.

Lastly, even though we have shown that the hybrid photocatalysts perform well when operating with the SiO_2_ coating, we should consider that the shell separates the components from the surface of Au. This impedes charge transfer, but it also limits the reaction’s enhancement through the near-field of the AuNP. This can be clearly seen by contrasting the AuNS and AuNS@SiO_2_ curves in Figure 4e or the electric field maps in Figure 4f,g. Consequently, one ought to consider the thickness of such insulating layers carefully, as it is a design parameter with a large direct impact on the performance of the photocatalyst. Both theoretical^63^ and experimental^37,64,65^ works have suggested that insulating layers of around 10 nm over small PNPs can provide a good balance between reducing dissipation mechanisms and still allowing enhancement through near-field interactions, with larger particles,^37^ films,^66^ and structured metamaterials^67^ having shown optima at larger thicknesses. Additionally, we present in Figure S4 a comparison of AuNRs coated with different SiO_2_ thicknesses, showing how insulating layers above ∼10 nm block the interaction between the PNPs and TiO_2_ in RhB photodegradation experiments. On the other hand, SiO_2_ layers below ∼8 nm in thickness often have less regular shapes, introducing variability in the barrier thickness.

## Conclusions

We have refined a remarkable hierarchical layer-by-layer assembly protocol for the creation of advanced photocatalytic hybrid nanostructures. Through the strategic combination of PNPs with CdS-QDs, we have achieved an unprecedented enhancement of the photocatalytic activity displayed by TiO_2_ NPs. Within this catalyst family, the CdS-QDs effectively utilize the near-field enhancement of the metal, underscoring the significance of the spectral overlap between plasmons and semiconductors. In fact, in pursuing the optimization of these hybrid systems for photocatalysis, we have found that focusing on the near-field enhancement of the CdS-QDs, bridging the energy transfer from SiO_2_-coated PNPs to TiO_2_ with an indirect charge transfer initiated in the QDs, yields the largest reaction rates. These rates surpass those arising from combining, using naked PNPs, HEI, and closer-range near-field enhancement due to the deleterious contribution of charge back-transfer to the naked metal. To conclude our exploration of hybrid composition engineering, we compared different sequences of photocatalyst element deposition and demonstrated the crucial importance of selecting an order that maximizes the coverage of the PNP surface with CdS-QD@TiO_2_-NP groups. This is done to exploit the aforementioned indirect activation mechanism. Our study also explores systems in which near-field-initiated mechanisms coexist with hot carrier injection. The results obtained from hybrids containing uncoated PNPs demonstrate how selecting a PNP geometry that enhances hot carrier excitation rates—AuNRs in our study—becomes a superior overall photosensitizer. However, the competing effect of electron back-transfer renders this configuration suboptimal when using QDs as the intermediate photosensitizer. These results present a detailed picture of the interplay between the different energy transfer mechanisms involved in hybrid plasmonic photocatalytic systems and underscore the importance of further detailed nanoengineering studies. This opens a path toward the synthesis of complexes that can optimize the different contributions of each component.

## Methods

### Materials

Cadmium nitrate [99%, (Cd(NO_3_)_2_)], sodium sulfide (99%, NaS_2_), l-cysteine (99%), sodium hydroxide (98%, NaOH), sodium borohydride (NaBH_4_), cetyltrimethylammonium bromide (96%, CTAB), silver nitrate (AgNO_3_), hydrochloric acid (37%, HCl), tetrachloroauric acid (HAuCl_4_·3H_2_O), l-ascorbic acid (99%, AA), poly(allylamine hydrochloride) (MW 17,500, PAH), tetraethylorthosilicate (98%, TEOS), ammonium hydroxide solution (28%–30%, NH_4_OH), sodium citrate (Na_3_C_6_H_5_O_7_), sodium chloride (NaCl), rhodamine B (RhB), formic acid (99%, FA), and poly(styrenesulfonate) (MW 70,000, PSS) were purchased from Sigma-Aldrich. TiO_2_ nanoparticles 5 nm (anatase ≥99%) were purchased from Nanoamor. Milli-Q water and absolute ethanol were used in all preparations.

### Synthesis and PAH Functionalization of Silica Beads

Monodisperse silica spheres with a diameter of 520 nm were prepared using a modified Stöber method.^68^ Typically, a TEOS solution (1.7 mL, 1.2 M) was added to a solution containing ethanol (18.12 mL), ammonium hydroxide (1.96 mL), and water (3.21 mL). This mixture was stirred at room temperature for 2 h. The excess reagents were removed by three centrifugation–redispersion cycles with ethanol (5000 rpm, 15 min).

Subsequently, PAH was dissolved in a 0.5 M NaCl aqueous solution with a final polymer concentration of 1 mg/mL. Then, 30 mL of the positively charged PAH solution was added to 30 mg of silica nanoparticles and stirred at room temperature for 30 min. The excess reagents were removed by three centrifugation–redispersion cycles with water (5000 rpm, 15 min).

### Synthesis of Gold Nanospheres

50 mL of a stable dispersion of spherical negatively charged citrate-stabilized gold nanoparticles, or nanospheres (AuNSs), was prepared by a method described elsewhere.^69^ The final diameter was fixed at 23 nm ± 2 nm after two growth steps.

### Synthesis and PSS Functionalization of AuNRs

50 mL of AuNRs with LSPR centered at 856 nm was synthesized by the seed-mediated growth method, as described elsewhere.^70^ The dimensions obtained from the TEM analysis images were 54 nm ± 4 nm of length and 13 nm ± 1 nm of thickness. Then, 25 mL of the final CTAB-stabilized AuNR solution was subsequently coated with a layer of a negatively charged polyelectrolyte (PSS) in order to facilitate the deposition onto the positively charged PAH-functionalized silica beads.^71^

### Silica Coating of AuNSs and AuNRs

25 mL of the citrate-stabilized AuNS and 25 mL of the CTAB-stabilized AuNR were coated with a thin layer of silica by following a previously published procedure.^72^ The silica thickness was 8 nm ± 1 nm for AuNS@SiO_2_ and AuNR@SiO_2_ (Figure S1).

### Deposition of AuNS, AuNS@SiO_2_, AuNR@PSS, and AuNR@SiO_2_ onto PAH-Functionalized Silica Beads

0.5 mL of each solution of PNP (*C*_Au_ = 0.5 mM) was added to 5 mL of PAH-functionalized silica beads (1 mg/mL). The mixture was stirred at room temperature for 3 h and washed by three centrifugation–redispersion cycles (5000 rpm, 20 min). The product was re-dispersed in 5 mL of water and functionalized with another layer of PAH in order to deposit the cadmium sulfide QDs.

### Synthesis of Cadmium Sulfide Quantum Dots and Deposition onto SiO_2_-PNP Assemblies

CdS-QDs were prepared using a modification of the protocol reported by Bae et al.^73^ Under a N_2_ atmosphere, 1 mmol of l-cysteine and 0.5 mmol of cadmium nitrate, were placed in a flask and dissolved in 100 mL of Milli-Q water with the pH value adjusted at 7.0 by using a prober buffer solution. The mixture was kept under stirring at 47 °C. Then, a sodium sulfide solution (0.51 mmol) was added dropwise under continuous stirring and allowed to react for 2 h. The CdS-QD formation was confirmed by UV–vis spectroscopy (Figure 1d), and the solution was kept in the dark.

1 mL of the synthesized CdS-QDs (0.72 mg/mL) was added to 5 mL of each solution of the PAH-functionalized SiO_2_-PNP assemblies, and the mixture was stirred at room temperature for 3 h. The excess of QDs was removed by three centrifugation–redispersion cycles (5000 rpm, 20 min), and the SiO_2_-PNP-QDs assemblies were functionalized with another layer of PAH.

### TiO_2_ Deposition

50 mg of TiO_2_ (anatase, 5 nm nanoparticles) re-dispersed in 100 mL of a sodium citrate solution (2.5 mM) was sonicated for 1 h with an ultrasonic tip. The aggregates of TiO_2_ NPs were removed by centrifugation (3500 rpm, 10 min). Then, 5 mL of each solution of the PAH-functionalized SiO_2_-PNP-QD assemblies was added to 4 mL of the TiO_2_ solution, and the mixture was stirred for 3 h. The excess TiO_2_ was removed by three centrifugation–redispersion cycles (5000 rpm, 20 min), and the final catalysts were protected from light until the photocatalytic experiments. The final molar PNP/TiO_2_/CdS ratios for all the samples were determined by inductively coupled plasma mass spectrometry (ICP-OES, Table S1).

### Chemical, Structural, and Optical Characterization

TEM images were obtained using a JEOL JEM 1010 transmission electron microscope operating at an acceleration voltage of 100 kV. UV–visible–NIR absorbance spectra were recorded on a Cary 8454 UV–visible–NIR spectrophotometer fitted with a thermostat holder and collected from a 1 cm-path-length quartz cuvette. Quantitative element detection from liquid samples was performed using a PerkinElmer Optima 4300 ICP-OES spectrometer with the samples previously digested in hydrofluoric acid.

### Photocatalytic Studies

#### Photocatalytic Degradation of RhB

The photocatalytic activity of the nanohybrids was evaluated by the degradation of RhB in a magnetically stirred sample in a bath at 25 °C under light irradiation in a LOT solar simulator (300 W Xe lamp, wavelength excitation from 350 nm to 2400 nm). The study is carried out in a 20 mL aqueous solution with a concentration of RhB of 0.01 mM and 4 mg of the hybrid photocatalyst. The mixtures were stirred for 1 h in the dark to blend well and allow the adsorption–desorption equilibrium to take place before irradiation. Aliquots of 2.5 mL were taken within 30 min intervals during the experiments in order to measure the variation in the absorbance of the dye. The photocatalytic activity of the hybrids was measured in terms of photodegradation of RhB over irradiation time.

#### Photocatalytic Dehydrogenation of Formic Acid

Photocatalytic hydrogen generation assisted by formic acid has been followed using nanohybrids as photocatalysts. Typically, 5 mL of an aqueous dispersion of the catalyst (0.8 mg/mL) was mixed with 200 μL of formic acid in a 13 mL reactor. The gases were purged with Ar for 2 min before sealing the flask. The dispersion was magnetically stirred inside a water bath at 35 °C under light irradiation with the solar simulator (λ∈ [350, 2400] nm). After 1 h, the gases were analyzed with an Agilent 7820A gas chromatographer to measure the volume of H_2_ generated.

### Simulations

Theoretical modeling has been performed in order to allow a correct interpretation of the optical and photochemical data obtained experimentally. To do so, we have solved the classical electrodynamic response of the plasmonic particles using a solver that uses finite element methods (FEM), COMSOL Multiphysics, and from these results, we have derived the relevant contribution of the different systems to hot carrier injection and near-field absorption enhancement. For the latter, we have computed the average FE around the systems as
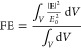
1where **E** is the electric field, *E*_0_ is the background field created by the incident plane wave and *V* is the volume surrounding the plasmonic material up to a distance of 20 nm from its surface. It is important to note that, in the case of SiO_2_-coated AuNP, the boundary of this volume remains unchanged but the volume filled with SiO_2_ is not included in *V* as we are interested in the effect of the near-field onto the absorption of TiO_2_ or the QDs. The permittivity of the materials was taken from experimental values and was assumed to be immersed in a medium with a dielectric constant *n* = 1.33. The permittivity of gold was broadened by a factor of 2 using the Drude model to account for crystal imperfections. The models for Au nanorods were 56 nm in length and 13 nm in diameter, and the Au spheres were 23 nm in diameter. In both cases, the models with a SiO_2_ layer had a thickness of 8 nm.

When it comes to computing the rate of excitation of intraband hot carriers at the surface of the plasmonic metal, we have used a formalism detailed in a previous work.^74^ In particular, we have used the expression
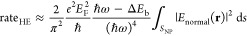
to compute the rate of excitation of hot carriers with excess energy above Δ*E* = 1 eV. This expression is derived from a quantum formalism but uses the local results of the electric field inside of the Au metal from the classical simulations. In the above expression, *E*_normal_(**r**) is the component of the field normal to the metal–environment interface, measured just inside the metal. The expression ℏω denotes the energy of the incoming photons, *E*_F_ is the Fermi energy of the metal, and *S*_NP_ is the surface of the metal particle. The intensity of the source of radiation was chosen as having a simple flat spectrum with an irradiance of *I*_0_ = 1.25 W/m^2^ per wavelength, chosen as a representative value of a typical solar irradiance at sea level in the visible range.

## Supporting Information Available

TEM images and size histograms of AuNS@SiO2 and AuNR@SiO2, details on Ti, Au, and Cd catalysts (in nanomoles per milligram of catalyst) for all the assemblies, photocatalytic degradation of RhB using a band-pass filter, additional computational results, and data showing the impact of SiO2 layer thickness (PDF)

## References

[ref1] Fujishima A.; Zhang X.; Tryk D. A. TiO2 Photocatalysis and Related Surface Phenomena. Surf. Sci. Rep. 2008, 63, 515–582. 10.1016/j.surfrep.2008.10.001.

[ref2] Sousa-Castillo A.; Couceiro J. R.; Tomás-Gamasa M.; Mariño-López A.; López F.; Baaziz W.; Ersen O.; Comesaña-Hermo M.; Mascareñas J. L.; Correa-Duarte M. A. Remote Activation of Hollow Nanoreactors for Heterogeneous Photocatalysis in Biorelevant Media. Nano Lett. 2020, 20, 7068–7076. 10.1021/acs.nanolett.0c02180. 32991175

[ref3] Chen J. J.; Wu J. C. S.; Wu P. C.; Tsai D. P. Plasmonic Photocatalyst for H2 Evolution in Photocatalytic Water Splitting. J. Phys. Chem. C 2011, 115, 210–216. 10.1021/jp1074048.

[ref4] Malato S.; Fernández-Ibáñez P.; Maldonado M. I.; Blanco J.; Gernjak W. Decontamination and Disinfection of Water by Solar Photocatalysis: Recent Overview and Trends. Catal. Today 2009, 147, 1–59. 10.1016/j.cattod.2009.06.018.

[ref5] Keane D. A.; McGuigan K. G.; Ibáñez P. F.; Polo-López M. I.; Byrne J. A.; Dunlop P. S. M.; O’Shea K.; Dionysiou D. D.; Pillai S. C. Solar Photocatalysis for Water Disinfection: Materials and Reactor Design. Catal. Sci. Technol. 2014, 4, 1211–1226. 10.1039/c4cy00006d.

[ref6] Afzal S.; Daoud W. A.; Langford S. J. Photostable Self-Cleaning Cotton by a Copper(II) Porphyrin/TiO2 Visible-Light Photocatalytic System. ACS Appl. Mater. Interfaces 2013, 5, 4753–4759. 10.1021/am400002k. 23465549

[ref7] Linic S.; Christopher P.; Ingram D. B. Plasmonic-Metal Nanostructures for Efficient Conversion of Solar to Chemical Energy. Nat. Mater. 2011, 10, 911–921. 10.1038/nmat3151. 22109608

[ref8] Zhang G.; Kim G.; Choi W. Visible Light Driven Photocatalysis Mediated via Ligand-to-Metal Charge Transfer (LMCT): An Alternative Approach to Solar Activation of Titania. Energy Environ. Sci. 2014, 7, 954–966. 10.1039/c3ee43147a.

[ref9] Devi L. G.; Kavitha R. A Review on Non Metal Ion Doped Titania for the Photocatalytic Degradation of Organic Pollutants under UV/Solar Light: Role of Photogenerated Charge Carrier Dynamics in Enhancing the Activity. Appl. Catal., B 2013, 140–141, 559–587. 10.1016/j.apcatb.2013.04.035.

[ref10] Dozzi M. V.; Selli E. Doping TiO2 with P-Block Elements: Effects on Photocatalytic Activity. J. Photochem. Photobiol., C 2013, 14, 13–28. 10.1016/j.jphotochemrev.2012.09.002.

[ref11] Sousa-Castillo A.; Comesaña-Hermo M.; Rodríguez-González B.; Pérez-Lorenzo M.; Wang Z.; Kong X. T.; Govorov A. O.; Correa-Duarte M. A. Boosting Hot Electron-Driven Photocatalysis through Anisotropic Plasmonic Nanoparticles with Hot Spots in Au-TiO2 Nanoarchitectures. J. Phys. Chem. C 2016, 120, 11690–11699. 10.1021/acs.jpcc.6b02370.

[ref12] Cushing S. K.; Li J.; Meng F.; Senty T. R.; Suri S.; Zhi M.; Li M.; Bristow A. D.; Wu N. Photocatalytic Activity Enhanced by Plasmonic Resonant Energy Transfer from Metal to Semiconductor. J. Am. Chem. Soc. 2012, 134, 15033–15041. 10.1021/ja305603t. 22891916

[ref13] Cushing S. K.; Li J.; Bright J.; Yost B. T.; Zheng P.; Bristow A. D.; Wu N. Controlling Plasmon-Induced Resonance Energy Transfer and Hot Electron Injection Processes in Metal@TiO2 Core-Shell Nanoparticles. J. Phys. Chem. C 2015, 119, 16239–16244. 10.1021/acs.jpcc.5b03955.

[ref14] Bumajdad A.; Madkour M. Understanding the Superior Photocatalytic Activity of Noble Metals Modified Titania under UV and Visible Light Irradiation. Phys. Chem. Chem. Phys. 2014, 16, 7146–7158. 10.1039/c3cp54411g. 24554000

[ref15] Ma X. C.; Dai Y.; Yu L.; Huang B. B. Energy Transfer in Plasmonic Photocatalytic Composites. Light: Sci. Appl. 2016, 5, e16017 10.1038/lsa.2016.17. 30167139 PMC6062428

[ref16] Vu N. N.; Kaliaguine S.; Do T. O. Plasmonic Photocatalysts for Sunlight-Driven Reduction of CO2: Details, Developments, and Perspectives. ChemSusChem 2020, 13, 3967–3991. 10.1002/cssc.202000905. 32476290

[ref17] Mascaretti L.; Naldoni A. Hot Electron and Thermal Effects in Plasmonic Photocatalysis. J. Appl. Phys. 2020, 128, 041101 10.1063/5.0013945.

[ref18] Baffou G.; Bordacchini I.; Baldi A.; Quidant R. Simple Experimental Procedures to Distinguish Photothermal from Hot-Carrier Processes in Plasmonics. Light: Sci. Appl. 2020, 9, 108 10.1038/s41377-020-00345-0. 32612818 PMC7321931

[ref19] Negrín-Montecelo Y.; Brissaud C.; Piquemal J. Y.; Govorov A. O.; Correa-Duarte M. A.; Besteiro L. V.; Comesaña-Hermo M. Plasmonic Photocatalysis in Aqueous Solution: Assessing the Contribution of Thermal Effects and Evaluating the Role of Photogenerated ROS. Nanoscale 2022, 14, 11612–11618. 10.1039/d2nr02431d. 35866634

[ref20] Rej S.; Santiago E. Y.; Baturina O.; Zhang Y.; Burger S.; Kment S.; Govorov A. O.; Naldoni A. Colloidal Titanium Nitride Nanobars for Broadband Inexpensive Plasmonics and Photochemistry from Visible to Mid-IR Wavelengths. Nano Energy 2022, 104, 107989 10.1016/j.nanoen.2022.107989.

[ref21] Rej S.; Mascaretti L.; Santiago E. Y.; Tomanec O.; Kment Š.; Wang Z.; Zbořil R.; Fornasiero P.; Govorov A. O.; Naldoni A. Determining Plasmonic Hot Electrons and Photothermal Effects during H2 Evolution with TiN-Pt Nanohybrids. ACS Catal. 2020, 10, 5261–5271. 10.1021/acscatal.0c00343.

[ref22] Richardson H. H.; Carlson M. T.; Tandler P. J.; Hernandez P.; Govorov A. O. Experimental and Theoretical Studies of Light-to-Heat Conversion and Collective Heating Effects in Metal Nanoparticle Solutions. Nano Lett. 2009, 9, 1139–1146. 10.1021/nl8036905. 19193041 PMC2669497

[ref23] Cortés E.; Besteiro L. V.; Alabastri A.; Baldi A.; Tagliabue G.; Demetriadou A.; Narang P. Challenges in Plasmonic Catalysis. ACS Nano 2020, 14, 16202–16219. 10.1021/acsnano.0c08773. 33314905

[ref24] Kale M. J.; Avanesian T.; Christopher P. Direct Photocatalysis by Plasmonic Nanostructures. ACS Catal. 2014, 4, 116–128. 10.1021/cs400993w.

[ref25] Jin R.; Charles Cao Y.; Hao E.; Métraux G. S.; Schatz G. C.; Mirkin C. A. Controlling anisotropic nanoparticle growth through plasmon excitation. Nature 2003, 425, 487–490. 10.1038/nature02020. 14523440

[ref26] Bhanushali S.; Mahasivam S.; Ramanathan R.; Singh M.; Harrop Mayes E. L.; Murdoch B. J.; Bansal V.; Sastry M. Photomodulated Spatially Confined Chemical Reactivity in a Single Silver Nanoprism. ACS Nano 2020, 14, 11100–11109. 10.1021/acsnano.0c00966. 32790283

[ref27] Movsesyan A.; Muravitskaya A.; Besteiro L. V.; Santiago E. Y.; Ávalos-Ovando O.; Correa-Duarte M. A.; Wang Z.; Markovich G.; Govorov A. O. Creating Chiral Plasmonic Nanostructures Using Chiral Light in a Solution and on a Substrate: The Near-Field and Hot-Electron Routes. Adv. Opt. Mater. 2023, 2300013 10.1002/adom.202300013.

[ref28] Negrin-Montecelo Y.; Movsesyan A.; Gao J.; Burger S.; Wang Z. M.; Nlate S.; Pouget E.; Oda R.; Comesana-Hermo M.; Govorov A. O.; Correa-Duarte M. A. Chiral Generation of Hot Carriers for Polarization-Sensitive Plasmonic Photocatalysis. J. Am. Chem. Soc. 2022, 144, 1663–1671. 10.1021/jacs.1c10526. 35073069

[ref29] Ávalos-Ovando O.; Santiago E. Y.; Movsesyan A.; Kong X. T.; Yu P.; Besteiro L. V.; Khorashad L. K.; Okamoto H.; Slocik J. M.; Correa-Duarte M. A.; Comesaña-Hermo M.; Liedl T.; Wang Z.; Markovich G.; Burger S.; Govorov A. O. Chiral Bioinspired Plasmonics: A Paradigm Shift for Optical Activity and Photochemistry. ACS Photonics 2022, 9, 2219–2236. 10.1021/acsphotonics.2c00445.

[ref30] Wang J.; Koo K. M.; Wang Y.; Trau M. Engineering State-of-the-Art Plasmonic Nanomaterials for SERS-Based Clinical Liquid Biopsy Applications. Adv. Sci. 2019, 6, 1900730 10.1002/advs.201900730. PMC6891916 31832306

[ref31] Phan-Quang G. C.; Han X.; Koh C. S. L.; Sim H. Y. F.; Lay C. L.; Leong S. X.; Lee Y. H.; Pazos-Perez N.; Alvarez-Puebla R. A.; Ling X. Y. Three-Dimensional Surface-Enhanced Raman Scattering Platforms: Large-Scale Plasmonic Hotspots for New Applications in Sensing, Microreaction, and Data Storage. Acc. Chem. Res. 2019, 52, 1844–1854. 10.1021/acs.accounts.9b00163. 31180637

[ref32] Brongersma M. L.; Halas N. J.; Nordlander P. Plasmon-Induced Hot Carrier Science and Technology. Nat. Nanotechnol. 2015, 10, 25–34. 10.1038/nnano.2014.311. 25559968

[ref33] Negrín-Montecelo Y.; Comesaña-Hermo M.; Khorashad L. K.; Sousa-Castillo A.; Wang Z.; Pérez-Lorenzo M.; Liedl T.; Govorov A. O.; Correa-Duarte M. A. Photophysical Effects behind the Efficiency of Hot Electron Injection in Plasmon-Assisted Catalysis: The Joint Role of Morphology and Composition. ACS Energy Lett. 2020, 5, 395–402. 10.1021/acsenergylett.9b02478.

[ref34] Negrín-Montecelo Y.; Comesaña-Hermo M.; Kong X. T.; Rodríguez-González B.; Wang Z.; Pérez-Lorenzo M.; Govorov A. O.; Correa-Duarte M. A. Traveling Hot Spots in Plasmonic Photocatalysis: Manipulating Interparticle Spacing for Real-Time Control of Electron Injection. ChemCatChem 2018, 10, 1561–1565. 10.1002/cctc.201702053.

[ref35] Negrín-Montecelo Y.; Kong X. T.; Besteiro L. V.; Carbó-Argibay E.; Wang Z. M.; Pérez-Lorenzo M.; Govorov A. O.; Comesaña-Hermo M.; Correa-Duarte M. A. Synergistic Combination of Charge Carriers and Energy-Transfer Processes in Plasmonic Photocatalysis. ACS Appl. Mater. Interfaces 2022, 14, 35734–35744. 10.1021/acsami.2c08685. 35913208

[ref36] Sakthivel S.; Shankar M. V.; Palanichamy M.; Arabindoo B.; Bahnemann D. W.; Murugesan V. Enhancement of Photocatalytic Activity by Metal Deposition: Characterisation and Photonic Efficiency of Pt, Au and Pd Deposited on TiO 2 Catalyst. Water Res. 2004, 38, 3001–3008. 10.1016/j.watres.2004.04.046. 15261537

[ref37] Torimoto T.; Horibe H.; Kameyama T.; Okazaki K. I.; Ikeda S.; Matsumura M.; Ishikawa A.; Ishihara H. Plasmon-Enhanced Photocatalytic Activity of Cadmium Sulfide Nanoparticle Immobilized on Silica-Coated Gold Particles. J. Phys. Chem. Lett. 2011, 2, 2057–2062. 10.1021/jz2009049.

[ref38] Hayashido Y.; Naya S. I.; Tada H. Local Electric Field-Enhanced Plasmonic Photocatalyst: Formation of Ag Cluster-Incorporated AgBr Nanoparticles on TiO2. J. Phys. Chem. C 2016, 120, 19663–19669. 10.1021/acs.jpcc.6b04894.

[ref39] Li K.; Hogan N. J.; Kale M. J.; Halas N. J.; Nordlander P.; Christopher P. Balancing Near-Field Enhancement, Absorption, and Scattering for Effective Antenna-Reactor Plasmonic Photocatalysis. Nano Lett. 2017, 17, 3710–3717. 10.1021/acs.nanolett.7b00992. 28481115

[ref40] Li J.; Cushing S. K.; Meng F.; Senty T. R.; Bristow A. D.; Wu N. Plasmon-Induced Resonance Energy Transfer for Solar Energy Conversion. Nat. Photonics 2015, 9, 601–607. 10.1038/nphoton.2015.142.

[ref41] Gao X. F.; Sun W. T.; Hu Z. D.; Ai G.; Zhang Y. L.; Feng S.; Li F.; Peng L. M. An Efficient Method to Form Heterojunction CdS/TiO2 Photoelectrodes Using Highly Ordered TiO2 Nanotube Array Films. J. Phys. Chem. C 2009, 113, 20481–20485. 10.1021/jp904320d.

[ref42] Peter L. M.; Riley D. J.; Tull E. J.; Wijayantha K. G. U. Photosensitization of Nanocrystalline TiO2 by Self-Assembled Layers of CdS Quantum Dots. Chem. Commun. 2002, 10, 1030–1031. 10.1039/b201661c. 12122649

[ref43] Bjelajac A.; Petrović R.; Nedeljković J. M.; Djokić V.; Radetić T.; Ćirković J.; Janaćković D. Ex-Situ Sensitization of Ordered TiO2 Nanotubes with CdS Quantum Dots. Ceram. Int. 2015, 41, 7048–7053. 10.1016/j.ceramint.2015.02.010.

[ref44] Maiti S.; Dana J.; Ghosh H. N. Correlating Charge-Carrier Dynamics with Efficiency in Quantum-Dot Solar Cells: Can Excitonics Lead to Highly Efficient Devices?. Chem.—Eur. J. 2019, 25, 692–702. 10.1002/chem.201801853. 29992637

[ref45] Nasir J. A.; Rehman Z. U.; Shah S. N. A.; Khan A.; Butler I. S.; Catlow C. R. A. Recent Developments and Perspectives in CdS-Based Photocatalysts for Water Splitting. J. Mater. Chem. A 2020, 8, 20752–20780. 10.1039/d0ta05834c.

[ref46] Yalavarthi R.; Mascaretti L.; Kudyshev Z. A.; Dutta A.; Kalytchuk S.; Zbořil R.; Schmuki P.; Shalaev V. M.; Kment Š.; Boltasseva A.; Naldoni A. Enhancing Photoelectrochemical Energy Storage by Large-Area CdS-Coated Nickel Nanoantenna Arrays. ACS Appl. Energy Mater. 2021, 4, 11367–11376. 10.1021/acsaem.1c02183.

[ref47] Zheng L.; Teng F.; Ye X.; Zheng H.; Fang X. Photo/Electrochemical Applications of Metal Sulfide/TiO2 Heterostructures. Adv. Energy Mater. 2020, 10, 1902355 10.1002/aenm.201902355.

[ref48] Zhao D.; Yang C. F. Recent Advances in the TiO2/CdS Nanocomposite Used for Photocatalytic Hydrogen Production and Quantum-Dot-Sensitized Solar Cells. Renewable Sustainable Energy Rev. 2016, 54, 1048–1059. 10.1016/j.rser.2015.10.100.

[ref49] Li J.; Cushing S. K.; Zheng P.; Senty T.; Meng F.; Bristow A. D.; Manivannan A.; Wu N. Solar Hydrogen Generation by a CdS-Au-TiO2 Sandwich Nanorod Array Enhanced with Au Nanoparticle as Electron Relay and Plasmonic Photosensitizer. J. Am. Chem. Soc. 2014, 136, 8438–8449. 10.1021/ja503508g. 24836347

[ref50] Kandi D.; Behera A.; Martha S.; Naik B.; Parida K. M. Quantum Confinement Chemistry of CdS QDs plus Hot Electron of Au over TiO2 Nanowire Protruding to Be Encouraging Photocatalyst towards Nitrophenol Conversion and Ciprofloxacin Degradation. J. Environ. Chem. Eng. 2019, 7, 102821 10.1016/j.jece.2018.102821.

[ref51] Zhao H.; Huang F.; Hou J.; Liu Z.; Wu Q.; Cao H.; Jing Q.; Peng S.; Cao G. Efficiency Enhancement of Quantum Dot Sensitized TiO2/ZnO Nanorod Arrays Solar Cells by Plasmonic Ag Nanoparticles. ACS Appl. Mater. Interfaces 2016, 8, 26675–26682. 10.1021/acsami.6b06386. 27648815

[ref52] Zhao W.; Liu J.; Deng Z.; Zhang J.; Ding Z.; Fang Y. Facile Preparation of Z-Scheme CdS–Ag–TiO2 Composite for the Improved Photocatalytic Hydrogen Generation Activity. Int. J. Hydrogen Energy 2018, 43, 18232–18241. 10.1016/j.ijhydene.2018.08.026.

[ref53] Swearer D. F.; Zhao H.; Zhou L.; Zhang C.; Robatjazi H.; Martirez J. M. P.; Krauter C. M.; Yazdi S.; McClain M. J.; Ringe E.; Carter E. A.; Nordlander P.; Halas N. J. Heterometallic Antenna-Reactor Complexes for Photocatalysis. Proc. Natl. Acad. Sci. U.S.A. 2016, 113, 8916–8920. 10.1073/pnas.1609769113. 27444015 PMC4987788

[ref54] Yalavarthi R.; Yesilyurt O.; Henrotte O.; Kment Š.; Shalaev V. M.; Boltasseva A.; Naldoni A. Multimetallic Metasurfaces for Enhanced Electrocatalytic Oxidations in Direct Alcohol Fuel Cells. Laser Photonics Rev. 2022, 16, 2200137 10.1002/lpor.202200137.

[ref55] Yalavarthi R.; Henrotte O.; Kment Š.; Naldoni A. Determining the Role of Pd Catalyst Morphology and Deposition Criteria over Large Area Plasmonic Metasurfaces during Light-Enhanced Electrochemical Oxidation of Formic Acid. J. Chem. Phys. 2022, 157, 114706 10.1063/5.0102012. 36137800

[ref56] Liz-Marzán L. M.; Giersig M.; Mulvaney P. Synthesis of Nanosized Gold-Silica Core-Shell Particles. Langmuir 1996, 12, 4329–4335. 10.1021/la9601871.

[ref57] Caruso F.; Caruso R. A.; Möhwald H. Nanoengineering of Inorganic and Hybrid Hollow Spheres by Colloidal Templating. Science 1998, 282, 1111–1114. 10.1126/science.282.5391.1111. 9804547

[ref58] Funston A. M.; Novo C.; Davis T. J.; Mulvaney P. Plasmon Coupling of Gold Nanorods at Short Distances and in Different Geometries. Nano Lett. 2009, 9, 1651–1658. 10.1021/nl900034v. 19271775

[ref59] Su K. H.; Wei Q. H.; Zhang X.; Mock J. J.; Smith D. R.; Schultz S. Interparticle Coupling Effects on Plasmon Resonances of Nanogold Particles. Nano Lett. 2003, 3, 1087–1090. 10.1021/nl034197f.

[ref60] Santiago E. Y.; Besteiro L. V.; Kong X.-T.; Correa-Duarte M. A.; Wang Z.; Govorov A. O. Efficiency of Hot-Electron Generation in Plasmonic Nanocrystals with Complex Shapes: Surface-Induced Scattering, Hot Spots, and Interband Transitions. ACS Photonics 2020, 7, 2807–2824. 10.1021/acsphotonics.0c01065.

[ref61] Enthaler S.; Von Langermann J.; Schmidt T. Carbon Dioxide and Formic Acid - The Couple for Environmental-Friendly Hydrogen Storage?. Energy Environ. Sci. 2010, 3, 1207–1217. 10.1039/b907569k.

[ref62] Selopal G. S.; Mohammadnezhad M.; Besteiro L. V.; Cavuslar O.; Liu J.; Zhang H.; Navarro-Pardo F.; Liu G.; Wang M.; Durmusoglu E. G.; Acar H. Y.; Sun S.; Zhao H.; Wang Z. M.; Rosei F. Synergistic Effect of Plasmonic Gold Nanoparticles Decorated Carbon Nanotubes in Quantum Dots/TiO2 for Optoelectronic Devices. Adv. Sci. 2020, 7, 2001864 10.1002/advs.202001864. PMC7578890 33101875

[ref63] Anger P.; Bharadwaj P.; Novotny L. Enhancement and Quenching of Single-Molecule Fluorescence. Phys. Rev. Lett. 2006, 96, 113002 10.1103/PhysRevLett.96.113002. 16605818

[ref64] Wang Z.; Gao W.; Wang R.; Shao J.; Han Q.; Wang C.; Zhang J.; Zhang T.; Dong J.; Zheng H. Influence of SiO2 Layer on the Plasmon Quenched Upconversion Luminescence Emission of Core-Shell NaYF4:Yb,Er@SiO2@Ag Nanocomposites. Mater. Res. Bull. 2016, 83, 515–521. 10.1016/j.materresbull.2016.06.035.

[ref65] Rohani S.; Quintanilla M.; Tuccio S.; De Angelis F.; Cantelar E.; Govorov A. O.; Razzari L.; Vetrone F. Enhanced Luminescence, Collective Heating, and Nanothermometry in an Ensemble System Composed of Lanthanide-Doped Upconverting Nanoparticles and Gold Nanorods. Adv. Opt. Mater. 2015, 3, 1606–1613. 10.1002/adom.201500380.

[ref66] Su Q.; Jiang C.; Gou D.; Long Y. Surface Plasmon-Assisted Fluorescence Enhancing and Quenching: From Theory to Application. ACS Appl. Bio Mater. 2021, 4, 4684–4705. 10.1021/acsabm.1c00320. 35007020

[ref67] Damm S.; Fedele S.; Murphy A.; Holsgrove K.; Arredondo M.; Pollard R.; Barry J. N.; Dowling D. P.; Rice J. H. Plasmon Enhanced Fluorescence Studies from Aligned Gold Nanorod Arrays Modified with SiO2 Spacer Layers. Appl. Phys. Lett. 2015, 106, 183109 10.1063/1.4919968.

[ref68] Stöber W.; Fink A.; Bohn E. Controlled Growth of Monodisperse Silica Spheres in the Micron Size Range. J. Colloid Interface Sci. 1968, 26, 62–69. 10.1016/0021-9797(68)90272-5.

[ref69] Bastús N. G.; Comenge J.; Puntes V. Kinetically Controlled Seeded Growth Synthesis of Citrate-Stabilized Gold Nanoparticles of up to 200 Nm: Size Focusing versus Ostwald Ripening. Langmuir 2011, 27, 11098–11105. 10.1021/la201938u. 21728302

[ref70] Scarabelli L.; Sánchez-Iglesias A.; Pérez-Juste J.; Liz-Marzán L. M. A “Tips and Tricks” Practical Guide to the Synthesis of Gold Nanorods. J. Phys. Chem. Lett. 2015, 6, 4270–4279. 10.1021/acs.jpclett.5b02123. 26538043

[ref71] Pastoriza-Santos I.; Pérez-Juste J.; Liz-Marzán L. M. Silica-Coating and Hydrophobation of CTAB-Stabilized Gold Nanorods. Chem. Mater. 2006, 18, 2465–2467. 10.1021/cm060293g.

[ref72] Fernández-López C.; Mateo-Mateo C.; Álvarez-Puebla R. A.; Pérez-Juste J.; Pastoriza-Santos I.; Liz-Marzán L. M. Highly Controlled Silica Coating of PEG-Capped Metal Nanoparticles and Preparation of SERS-Encoded Particles. Langmuir 2009, 25, 13894–13899. 10.1021/la9016454. 19591480

[ref73] Bae W.; Abdullah R.; Mehra R. K. Cysteine-Mediated Synthesis of CdS Bionanocrystallites. Chemosphere 1998, 37, 363–385. 10.1016/s0045-6535(98)00051-4.

[ref74] Besteiro L. V.; Kong X. T.; Wang Z.; Hartland G.; Govorov A. O. Understanding Hot-Electron Generation and Plasmon Relaxation in Metal Nanocrystals: Quantum and Classical Mechanisms. ACS Photonics 2017, 4, 2759–2781. 10.1021/acsphotonics.7b00751.

