# Supplementary material for: Balancing Near-Field Enhancement and Hot Carrier Injection: Plasmonic Photocatalysis in Energy-Transfer Cascade Assemblies
Source: ACS Photonics. 2023 Sep 6;10(9):3310–20. doi: 10.1021/acsphotonics.3c00733 (PMC10516266; doi:10.1021/acsphotonics.3c00733)
Supplement: Supplementary file 1 — ph3c00733_si_001.pdf [file ph3c00733_si_001.pdf]

Supporting information

Balancing near-field enhancement and hot carrier injection: Plasmonic photocatalysis in energy-transfer cascade assemblies

Yoel Negrín-Montecelo,[a] *Adbelrhaman Hamdeldein Ahmed Geneidy,*[b] *Alexander O. Govorov,*[c] *Ramon A. Alvarez-Puebla,*[a,d]* *Lucas V. Besteiro*[b]**and Miguel A. Correa-Duarte.*[b,e]*

[a] Department of Physical and Inorganic Chemistry, Universitat Rovira i Virgili, Carrer de Marcel·lí Domingo s/n, 43007 Tarragona, Spain
[b] CINBIO, University of Vigo, Campus Universitario de Vigo, Lagoas Marcosende, 36310 Vigo, Spain
[c] Department of Physics and Astronomy, Ohio University, Athens, Ohio 45701, United States
[d] ICREA, Passeig Lluís Companys 23, 08010 Barcelona, Spain
[e] Southern Galicia Institute of Health Research (IISGS) and Biomedical Research Networking Center for Mental Health (CIBERSAM), Universidade de Vigo, 36310 Vigo, Spain

* ramon.alvarez@urv.cat, lucas.v.besteiro@uvigo.es, macorrea@uvigo.es

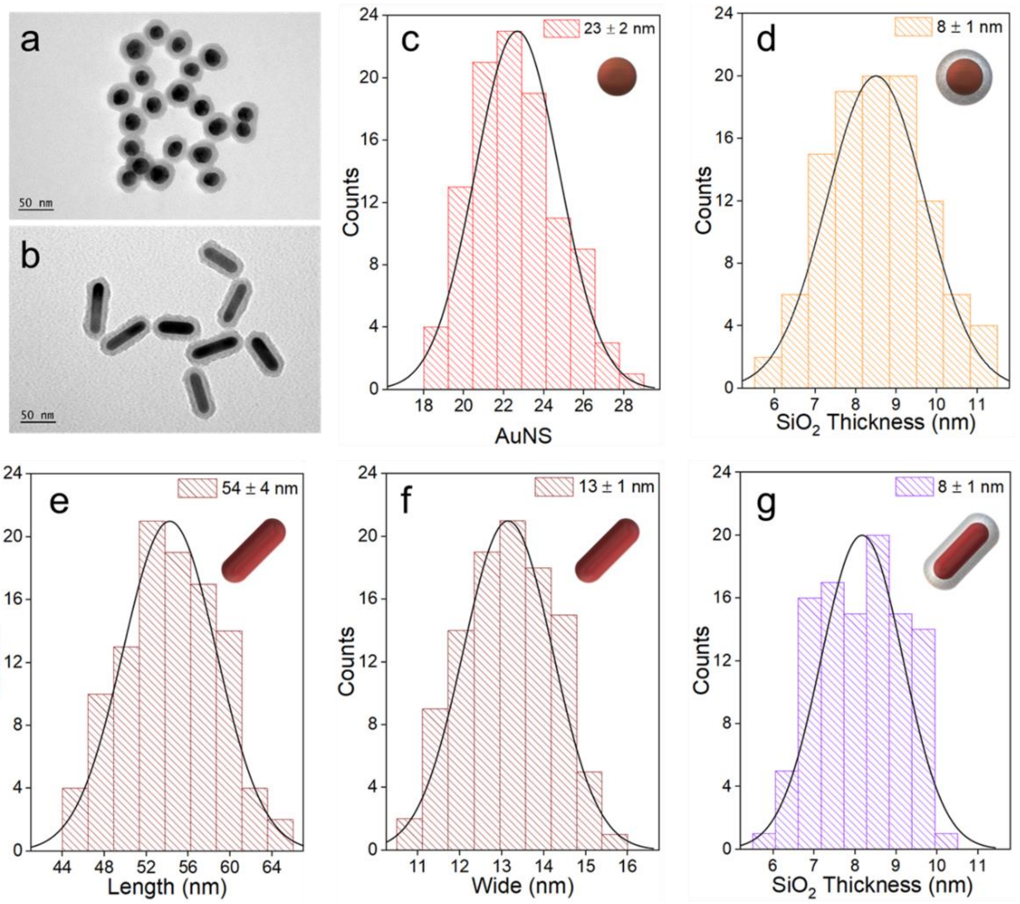

Figure S1. (a,b) TEM images of the AuNS@SiO_2 and AuNR@SiO_2, respectively. (c,d) Size histograms of core and shell in the AuNS@SiO_2. (e-g) Size histograms of core and shell in the AuNR@SiO_2.

**Sample: (SiO_2 bead with)**	**Ti (nmol/mg)**	**Au (nmol/mg)**	**Cd (nmol/mg)**
**TiO_2**	449,16	-	-
**CdS/TiO_2**	465,87	-	60,49
AuNS/TiO_2	460,19	30,85	-
AuNS@SiO_2/TiO_2	450,64	29,92	-
AuNR/TiO_2	451,25	33,00	-
AuNR@SiO_2/TiO_2	463,38	31,96	-
**AuNS/CdS/TiO_2**	457,56	35,77	51,35
**AuNS@SiO_2/CdS/TiO_2**	467,96	33,50	49,82
**AuNR/CdS/TiO_2**	453,34	33,00	59,60
**AuNR@SiO_2/CdS/TiO_2**	461,70	32,49	56,04

Table S1. Nanomoles of Ti, Au and Cd per milligram of catalyst for all the assemblies. These values were obtained by ICP-OES. The color code in the sample description follows that of the figures in the main text.

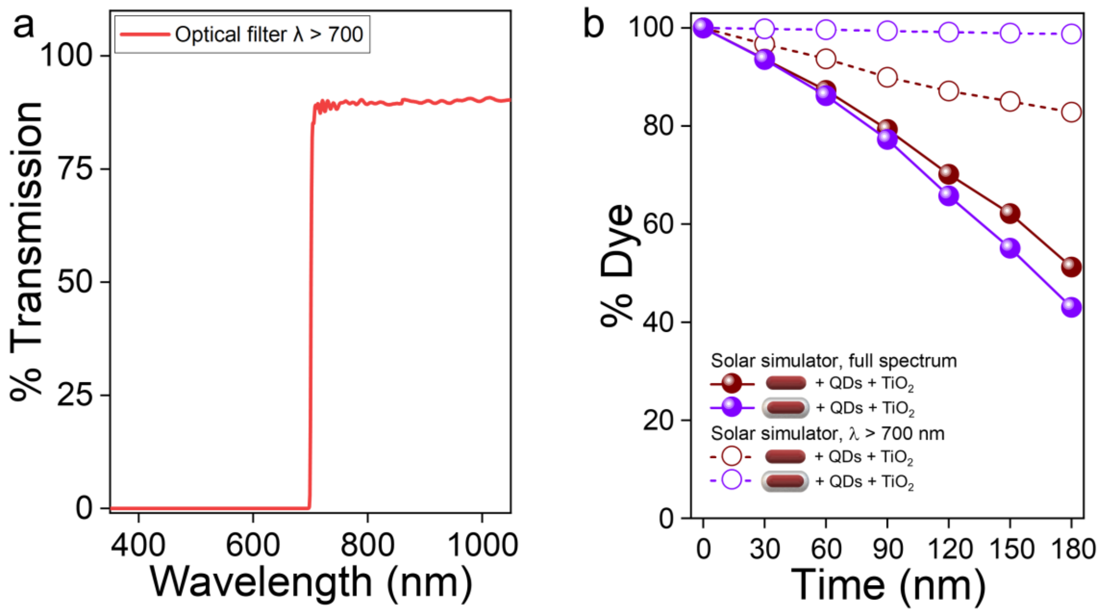

Figure S2. Photocatalytic degradation of RhB using a long-wavelength-pass filter. (a) Transmission spectrum of the filter. Its profile is remarkably flat from 700 nm on, although one has to note that its transmission is only around 90%, a factor to account for in analyzing degradation results. (b) Photocatalytic degradation of RhB for hybrids containing AuNRs with and without SiO_2 layer, contrasting the evolution of the reaction under the full spectrum of the solar simulator without the visible and UV light. The results under full spectrum illumination are those from Figure 3b.

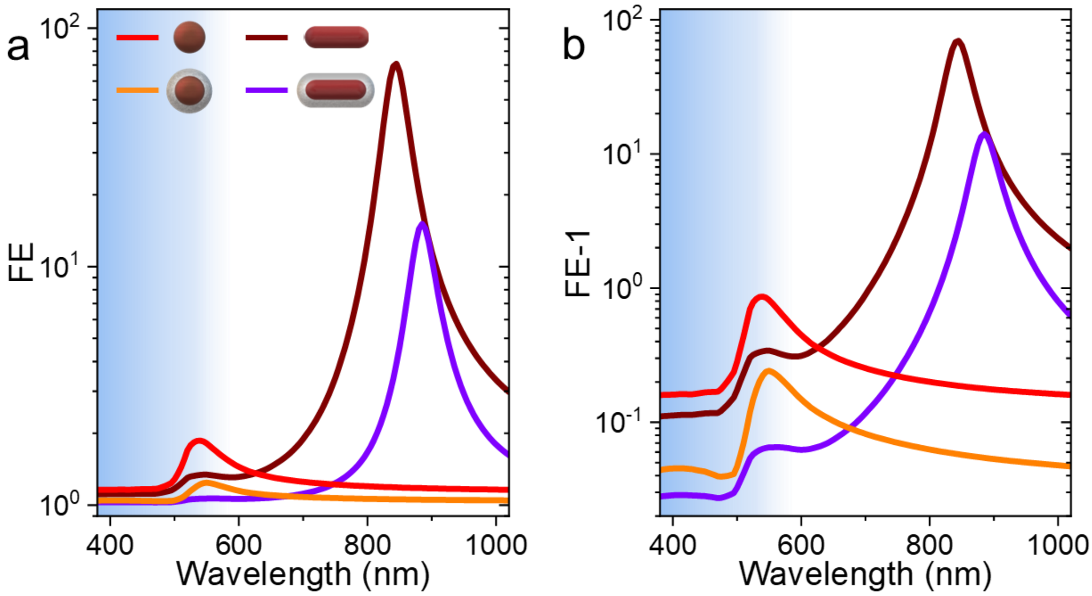

Figure S3. Computational results for the different plasmonic nanoparticles used in this study. (a) Volume-averaged FE (see eq. 1 in the main text). (b) Increase on the average field enhancement, over a system without PNP. The blue-shaded area indicates the spectral region with non-zero QD absorption. Contrasting with the data in Figure 4e in the main text, these curves correspond to values per-PNP, instead of "per-atom".

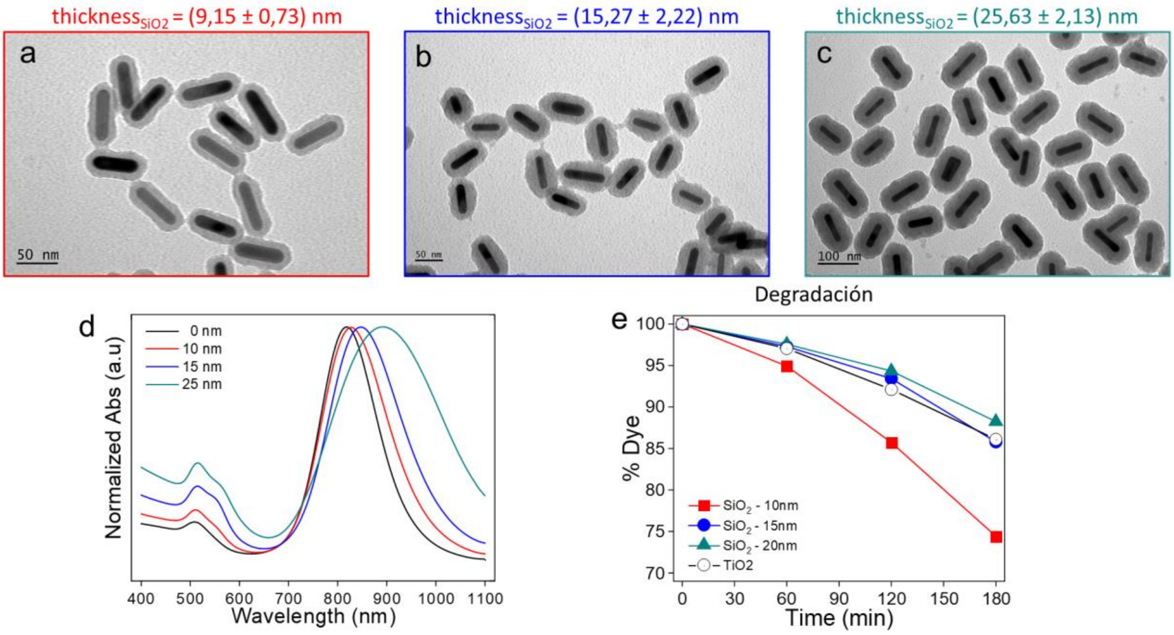

Figure S4. Testing the impact of SiO_2 layer thickness. (a-c) TEM images of AuNRs with increasing SiO_2 thickness. (d) Normalized absorbance of the AuNRs@SiO_2 of the samples shown in the top panels. Increasing the thickness of the SiO_2 layer increases the redshift of the longitudinal plasmonic mode and introduces a secondary transversal mode. (d) RhB degradation results with hybrids composed by SiO_2 beads and loaded with AuNRs@SiO_2 and TiO_2 NPs. Rods covered with SiO_2 layers thicker than ~10 nm do not enhance the degradation obtained with TiO_2 alone.
